# An intelligent grasper to provide real-time force feedback to shorten the learning curve in laparoscopic training

**DOI:** 10.1186/s12909-024-05155-1

**Published:** 2024-02-20

**Authors:** Xuemei Huang, Pingping Wang, Jie Chen, Yuxin Huang, Qiongxiu Liao, Yuting Huang, Zhengyong Liu, Dongxian Peng

**Affiliations:** 1 Obstetrics and Gynecology Center, Department of Gynecology, Zhujiang Hospital, Southern Medical University, Guangzhou, 510280 China; 2 Guangdong Provincial Key Laboratory of Optoelectronic Information Processing Chips and Systems, School of Electronics and Information Technology, Sun Yat-Sen University, Guangzhou, 510275 China

**Keywords:** Minimally invasive surgery, Fiber Bragg grating, Force feedback, Laparoscopic training, Learning curve

## Abstract

**Background:**

A lack of force feedback in laparoscopic surgery often leads to a steep learning curve to the novices and traditional training system equipped with force feedback need a high educational cost. This study aimed to use a laparoscopic grasper providing force feedback in laparoscopic training which can assist in controlling of gripping forces and improve the learning processing of the novices.

**Methods:**

Firstly, we conducted a pre-experiment to verify the role of force feedback in gripping operations and establish the safe gripping force threshold for the tasks. Following this, we proceeded with a four-week training program. Unlike the novices without feedback (Group A_2_), the novices receiving feedback (Group B_2_) underwent training that included force feedback. Finally, we completed a follow-up period without providing force feedback to assess the training effect under different conditions. Real-time force parameters were recorded and compared.

**Results:**

In the pre-experiment, we set the gripping force threshold for the tasks based on the experienced surgeons’ performance. This is reasonable as the experienced surgeons have obtained adequate skill of handling grasper. The thresholds for task 1, 2, and 3 were set as 0.731 N, 1.203 N and 0.938 N, respectively. With force feedback, the gripping force applied by the novices with feedback (Group B_1_) was lower than that of the novices without feedback (Group A_1_) (*p* < 0.005). During the training period, the Group B_2_ takes 6 trails to achieve gripping force of 0.635 N, which is lower than the threshold line, whereas the Group A_2_ needs 11 trails, meaning that the learning curve of Group B_2_ was significantly shorter than that of Group A_2_. Additionally, during the follow-up period, there was no significant decline in force learning, and Group B_2_ demonstrated better control of gripping operations. The training with force feedback received positive evaluations.

**Conclusion:**

Our study shows that using a grasper providing force feedback in laparoscopic training can help to control the gripping force and shorten the learning curve. It is anticipated that the laparoscopic grasper equipped with FBG sensor is promising to provide force feedback during laparoscopic training, which ultimately shows great potential in laparoscopic surgery.

## Introduction

In the past few decades, Minimally Invasive Surgery (MIS) has changed the surgical process [1–3], greatly improving the implementation of MIS in general surgery, gynecology, cardiothoracic surgery, colorectal surgery and urological surgery [4–7]. MIS has many advantages, such as small wound, quickly recovery, etc [1, 8]. However, the lack of force feedback, even completely lose in Robot-assisted minimally invasive surgery (RMIS), during operation restricts its development [9] and affects the training of novices’ operating during MIS to some extent. The lack of force feedback in MIS leads novices to undergo more training before operating successfully on patients [2]. Generally, the skill acquisition involves a steep learning curve [10]. Most residents who receive training in laparoscopic surgical skills take more than 3 years to master the operation skills [11]. Performing complex surgical tasks in laparoscopic surgery requires more precise control and extensive training [12].

Indeed, in order to meet the precise requirements of MIS, more and more simulation training and education methods outside of the operation room are proposed to improve the surgical skills of surgeons, particularly novice surgeons. With the increasing availability and use of laparoscopic training models, more and more novice surgeons are able to acquire the necessary skills for MIS through simulation-based training programs. This has the potential to improve patient outcome by reducing the risk of surgical errors and complications during actual procedures. There have been a series of training models developed for simulation-based surgical training. Currently, the most widely used simulation methods can be classified into three categories: box training (BT), virtual reality (VR) and augmented reality (AR) training. However, traditional BT models do not provide force feedback, which limits their ability to provide a realistic training context and objective results. On the other hand, VR training is able to provide objective results, but these results are typically not available to operators in real-time. AR that provides both haptic feedback and objective results during training [13] is costly. Therefore, it is essential to develop cost-effective training systems that can provide force feedback in order to enhance the effectiveness and realism of surgical simulation training. There are some systems appearing, which however rely on external force measuring platform and may lead to imprecise results [14]. For example, Luis et al. [15] used an intelligent trainer equipped with a gripping sensor to measure the gripping force, but the measuring force is not really accurate. Hardon et al. [16] use a box trainer with a built-in force tracking system to monitor the force to assess the operation skill of residents. In fact, due to the status of the usage of the training system, the training in MIS offering to novices is limited [17]. Regarding the current laparoscopic training system with force feedback, its high cost of training and absence of objective real-time assessments have resulted in a prolonged learning curve for novice surgeons [18]. Therefore, there is an urgent need to develop an effective and costless training system. In the training processing of MIS, it is preferable to use a laparoscopic grasper with real-time force feedback to achieve targeted training and increase the efficiency of every surgical operation. The training process will be effectively standardized and the training period will be shortened [19].

In the past few years, fiber Bragg grating (FBG) sensors have been widely applied in minimally invasive surgery to offer force feedback because of their small size, high sensitivity, good biocompatibility, light weight, immunity to electromagnetic interference (EMI), etc [20–24]. The surgical instrument integrated with FBG sensor provides the surgeon with force information during MIS, facilitating more precise and accurate operations. For example, Li et al. [25] proposed a three-axis tactile probe based on fiber grating, which can accurately identify long blood vessels in the prosthesis and locate the wrapped tissue in three dimensions. Furthermore, they verified its effectiveness and feasibility in isolated porcine kidney tissue. Xue et al. [26] introduced FBGs to grooves in the laparoscopic surgery robot, which is used to estimate gripping force and perform precise force control. Besides, Imbrie-moore et al. [27] mounted a pig mitral valve in a cardiac simulator, each valve was repaired with Teflon sutures. In their work, an FBG sensor was used to measure real-time suture force. In addition, Scott et al. [28] developed an FBG-based sensor and measured the force at the tip of the electrode array during insertion into the cochlea in real time in guinea pigs. Although FBG has been proposed for use in medical surgery, the additional value of force feedback based on FBG in MIS training has not been established.

In our previous study, we designed an intelligent laparoscopic grasper integrated with an FBG-based tactile sensor, which can provide real-time force feedback to the novice operators and has shown excellent performance in the laparoscopic training box [29]. As a continuation of this work, we utilized the laparoscopic grasper to provide real-time force feedback to the trainers during laparoscopic training, allowing for quantification of force information obtained during training. Results indicate that training system with an FBG force sensor has significant potential to shorten the learning curve of laparoscopic training by providing real-time force feedback to trainees through an intelligent laparoscopic grasper.

## Methods

The procedures, methods, and consent forms employed in this study received approval from the Ethics Review Board of Zhujiang Hospital, Southern Medical University. The training program, which was based on basic laparoscopic skills (FLS) closely simulated real-world clinical circumstance.

### The proposed laparoscopic training system and gripping tasks setting

**Fig. 1:**
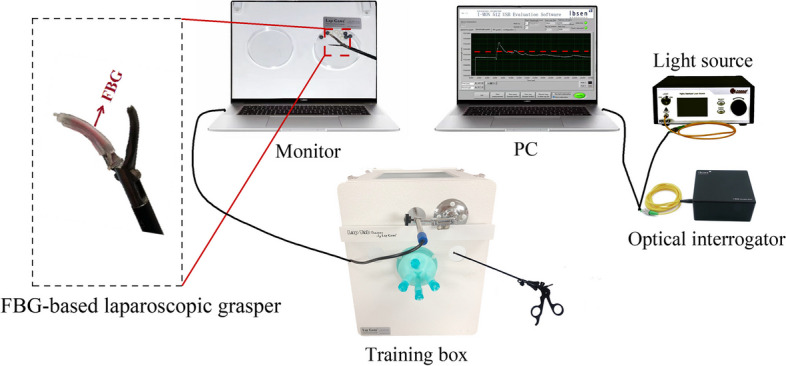
Instrument connection diagram

The proposed training system is shown in Fig. 1. To imitate the laparoscopic surgery, we use a validated Lap Game box trainer (Lap Game Inc., Hangzhou, China). The device (Fig. 1) includes a light source, an internal camera for imaging and a display screen for operation on the computer. Different from traditional laparoscopic training box, we used a previously designed smart laparoscopic grasper with fiber Bragg grating sensor during training [29]. The intelligent laparoscopic grasper is used to clamp the specific objects and the real-time force information obtained by the fiber Bragg grating sensor was demodulated by an optical spectrometer (I-MON 51 USB, Ibsen Photonics, Denmark). In principle, FBG can reflect a specific wavelength (also called Bragg wavelength λ _B_) of broadband light , which is determined by the effective index of refraction (n_eff_) of the fundamental mode propagating in the fiber core and the grating period (Λ), as expressed in Eq. (1). When there is a certain force applied to the FBG, the Bragg wavelength would show a corresponding shift due to the deformation of the grating as well as a change in the refractive index. This relationship can be defined by Eq. (2), in which *P*_*e*_ is photo-elastic coefficient, η is a factor of force transferred to strain and F is the applied force.

1 $${\uplambda}_B=2{n}_{\textrm{eff}}\Lambda$$

2 $$\Delta {\uplambda}_B=2{n}_{\textrm{eff}}\Lambda \left(1+{P}_e\right)\cdot \left(\upeta F\right)$$

In our study, we can obtain the gripping force by demodulating the Bragg wavelength change in the reflection spectrum detected by the spectrometer during the operation process. The real-time force information is displayed and stored on the computer.

The experiment consisted of 3 gripping transfer tasks (Table 1). These tasks are designed based on basic laparoscopic skills (FLS) training [30] and close to real clinical circumstance [31].

**Table 1:**
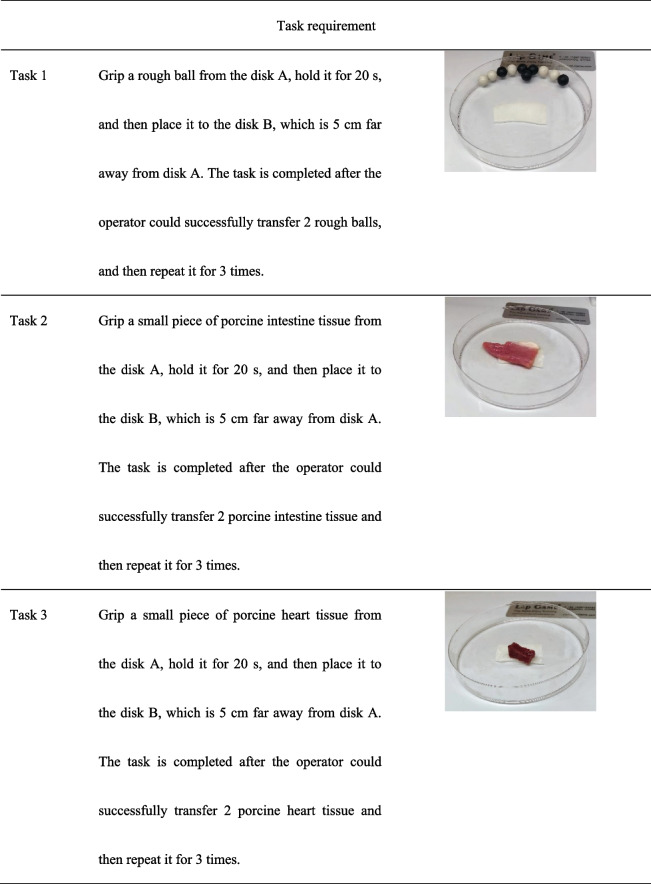
Gripping tasks setting

### Participant selection and baseline characteristics

We recruited medical students with no prior training in laparoscopic surgery from our institution’s medical school through a virtual announcement. Gynecologists with extensive experience (having perform over 100 advanced procedures) were selected as surgeons with experience for the study. During the study, the participants included 6 experienced surgeons and 42 novices. Novices were divided randomly into novices without force feedback (Group A) and novices with force feedback (Group B). All participants filled out the personal questionnaire before the experiment. The basic information of all subjects was documented and recorded in Table 2. In order to ensure that the novice participants had a similar level of laparoscopic surgery skills, 42 novices were required to complete Task 1 without feedback for baseline assessment. Novices at the same level were included in this study.

**Table 2:** Personal information of the participants

Baseline characteristics	Group A:Novices without force feedback (*n* = 21)	Group B:Novices with force feedback (*n* = 21)	Group C: Experienced without force feedback (*n* = 6)
Age (years)	24.19 ± 1.12	24.71 ± 1.38	34.67 ± 2.86
Gender	Female:16, male:5	Female:18, male:3	Female:4, male:2
Length of laparoscopic experiences	0	0	> 5
Number of laparoscopic operations	0	0	≥100
Dominant hand	Right:21, left:0	Right:21, left:0	Right:6, left:0
Laparoscopic feel, median (range)	(3.5,1–6)	(3.5,1–6)	(6.5,5–8)
Play video game (yes)	9	9	3

### Study protocol

#### Preliminary experiment

We conducted a pre-experiment to verify the role of force feedback in laparoscopic gripping and set the threshold for the gripping tasks. Without providing force feedback, the experienced surgeons (Group C) and six novices of Group A (Group A_1_) were asked to complete three tasks to see whether there is some difference between the two and further determine the threshold of the tasks. The threshold of the task was defined as the force level of safe grasping. We set the average of the maximum gripping force of the experienced surgeons as the threshold of the tasks. The thresholds for task 1, task 2 and task 3 are 0.731 N, 1.203 N and 0.938 N respectively. Then, with real-time force feedback, six novices of Group B (Group B_1_) carried out three tasks. Different from novices without force feedback (Group A_1_), novices with force feedback (Group B_1_) conducted the tasks with real-time force feedback, meaning that novices with force feedback can quantify the grasping force during the tasks and adjust the force they used according to the threshold of the tasks. Throughout the operation, all the grasping data was collected by the computer. A comparison of the grasping force between Group A_1_ and Group B_1_ was conducted. The purpose of the preliminary experiment is to determine whether force feedback during gripping operations can have a positive influence on MIS. Finally, laparoscopic training sessions were conducted for the remaining novices in the laparoscopic training box.

#### Training program

The other thirty novices who are at the same level in laparoscopic surgery entered the laparoscopic training. They are Group A_2_ and Group B_2_, i.e., the other 15 novices of Group A and Group B, respectively. A four-week laparoscopic training was conducted. Taking schedule of the novices into consideration, we ensured that all novices completed an equal number of gripping trials during this training period. In our study, 30 novices were assigned to conduct 10 trials at different time intervals. Each trial required them to successfully complete the task three times. It is important to note that all novices utilized the same smart laparoscopic grasper. As for the Group B_2_, with the real-time force feedback, participants were able to quantitatively measure the force applied during tasks and adjust it in time according to the predefined threshold. In contrast, Group A_2_ completed the training without force feedback, relying solely on subjective perception for force adjustment. All the grasping force data during the whole training was collected by the FBG force sensor.

#### Follow-up test

**Fig. 2:**
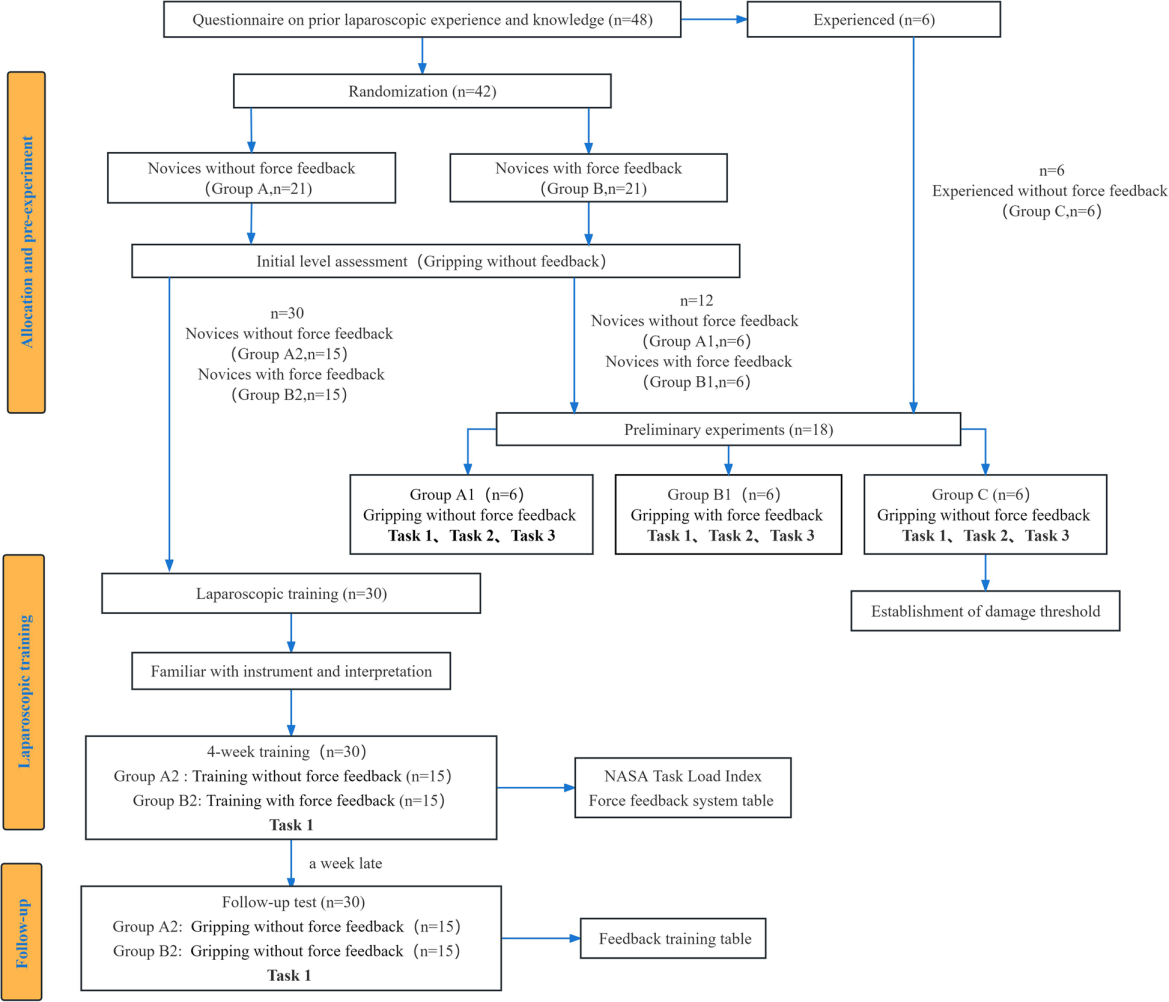
Schematic flowchart of the study protocol

Follow-up testing was carried out 1 week after the completion of training to evaluate the retention of grasping skills acquired during the training period. Both Group A_2_ and Group B_2_ were require to complete the task without force feedback in the follow-up period. Besides, all participants were asked to complete the NASA Task Load Index [32]. Only Group B_2_ filled out the Force Feedback System Evaluation Survey additionally. Figure 2 shows the schematic flowchart of the study protocol.

#### Outcome evaluation

The maximum absolute force and the standard deviation of the gripping force collected during the experiment are utilized to evaluate the effectiveness of the training. Additionally, scales filled out by the participants are analyzed at the same time to assess the training system from subjective point of view. A detailed overview and description of these parameters are provided in Table 3. In addition, in order to better analyze the control and learning progress of two groups during the training period, we plotted individual learning curves for each group and conducted a comparison analysis. Specifically, we recorded the maximum and standard deviation of the gripping force during 10 laparoscopic training sessions of novices under different feedback conditions. Additionally, we included the final follow-up stage where both groups performed gripping operations without force feedback. The training curves were plotted at each data point. Drawing the learning curves during the laparoscopic training allows for a more intuitive observation of the impact of real-time force feedback. All participants were asked to fill out a questionnaire and NASA Task Load Index after completion of the four-week training to obtain information about their general impression of the force feedback system and training tasks. General comments on the training were obtained from participants and presented using a 5 points Likert scale.

**Table 3:** Description of outcome evaluation

Evaluation index	Description
Maximum absolute force (Newton)	The highest absolute force during the measurement, which may cause tissue damage in the laparoscopic surgery.
SD of absolute force (Newton)	The standard deviation of the absolute force that can reflect the stability during the gripping tasks.
Force feedback system evaluation scale	The scale used to evaluate the satisfaction of the design of the system equipped with force feedback.
Training feedback evaluation scale	The scale used to evaluate the training effect by self-rating, which involves skills improving, operating confidence, etc.
NASA Task Load Index	The scale includes questions of the participants’ operating feeling during the training.

Data were analyzed using IBM SPSS statistical version 23.0 (IBM Corp, Armonk, NY). The t-test was employed for normally distributed data, while nonparametric Mann-Whitney U test was used for non-normally distributed data. Basically, a probability of *p*<0.05 was considered statistically significant [33].

## Results

### Baseline assessment of the novices

**Fig. 3:**
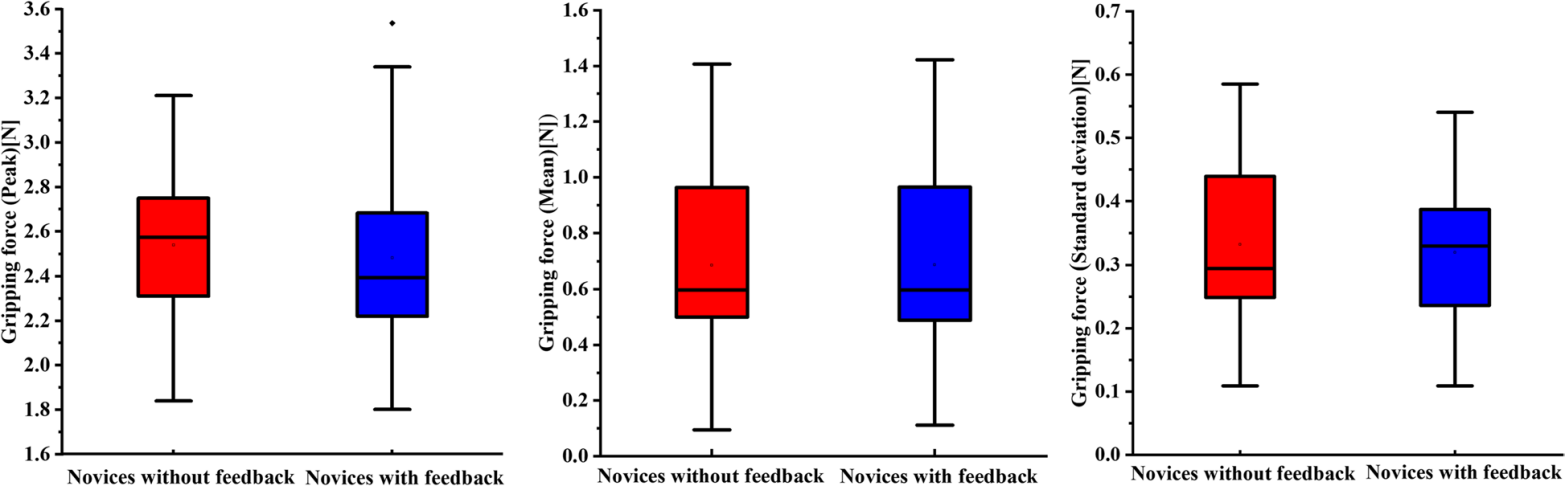
Box plots of the gripping force among novices for baseline assessment

At the baseline evaluation, all novices had no prior experience in laparoscopic operation. Figure 3 shows the box plots of gripping force that represents the baseline assessment of the novices (*n* = 42). Obviously, none of the peak, mean and standard deviation of the gripping force showed a significant difference among the novices, indicating that the two groups of novices were at the same level of laparoscopic grasping (*p =* 0.653, 0.996, 0.831 respectively).

### Analysis results of preliminary experimental

In the preliminary experiments, Table 4 presents the gripping force of three groups in different tasks. The statistical results comparing the three groups are also summarized. Taking task 1 as an example, the average of the maximum gripping force for experienced surgeons was 0.731 N, while novices without feedback recorded 3.686 N. The standard deviation of the gripping force for the two groups was 0.096 and 0.468 respectively. The force values for the task exhibited significantly differences (*p* < 0.001). The results indicated that the experienced surgeons exhibited significantly lower force and better stability of control than the novices without feedback. Similar results were observed across other tasks. Therefore, we established the average maximum value of the experienced surgeons as the threshold for the tasks. The threshold for task 1, task 2 and task 3 were determined to be 0.731 N, 1.203 N and 0.938 N respectively. With providing real-time force feedback to Group B_1_, the maximum and the standard deviation of the gripping force of task 1 is 0.979 N and 0.112, while is 3.686 N and 0.486 of Group A_1_. In the comparison between Group A_1_ and Group B_1_, the force values of all tasks were compared, resulting in a *p* value of less than 0.05. This indicates that the introduction of force feedback has obvious advantages in better maintaining gripping force. Additionally, the maximum value of all tasks in novices with feedback and experienced surgeons is significantly different(*p*<0.001). Although novices with feedback exhibit better control of gripping force, this result suggests that, compared to the experienced surgeons, novices still have room for improvement in controlling gripping force. Therefore, implementing a standardized training process is necessary to further enhance the control of gripping force.

**Table 4:** Statistical results between different groups in all the tasks

	Group A_1_: Novices without feedback	Group B_1_: Novices with feedback	Group C: Experienced without feedback	*P* value	A_1_-B_1_	B_1_-C	A_1_-C
Task 1							
Peak force	3.686	0.979	0.731		*< 0.001*	*0.001*	*< 0.001*
Standard deviation of force	0.468	0.112	0.096		*< 0.001*	0.264	*< 0.001*
Task 2							
Peak force	4.749	1.876	1.203		*< 0.001*	*< 0.001*	*< 0.001*
Standard deviation of force	0.839	0.242	0.132		*0.005*	*< 0.001*	*< 0.001*
Task 3							
Peak force	4.114	1.315	0.938		*< 0.001*	*< 0.001*	*< 0.001*
Standard deviation of force	0.698	0.161	0.113		*< 0.001*	*0.002*	*< 0.001*

Significant *p* values are given in italics (*p* < 0.05)

### Analysis results of the training program

**Fig. 4:**
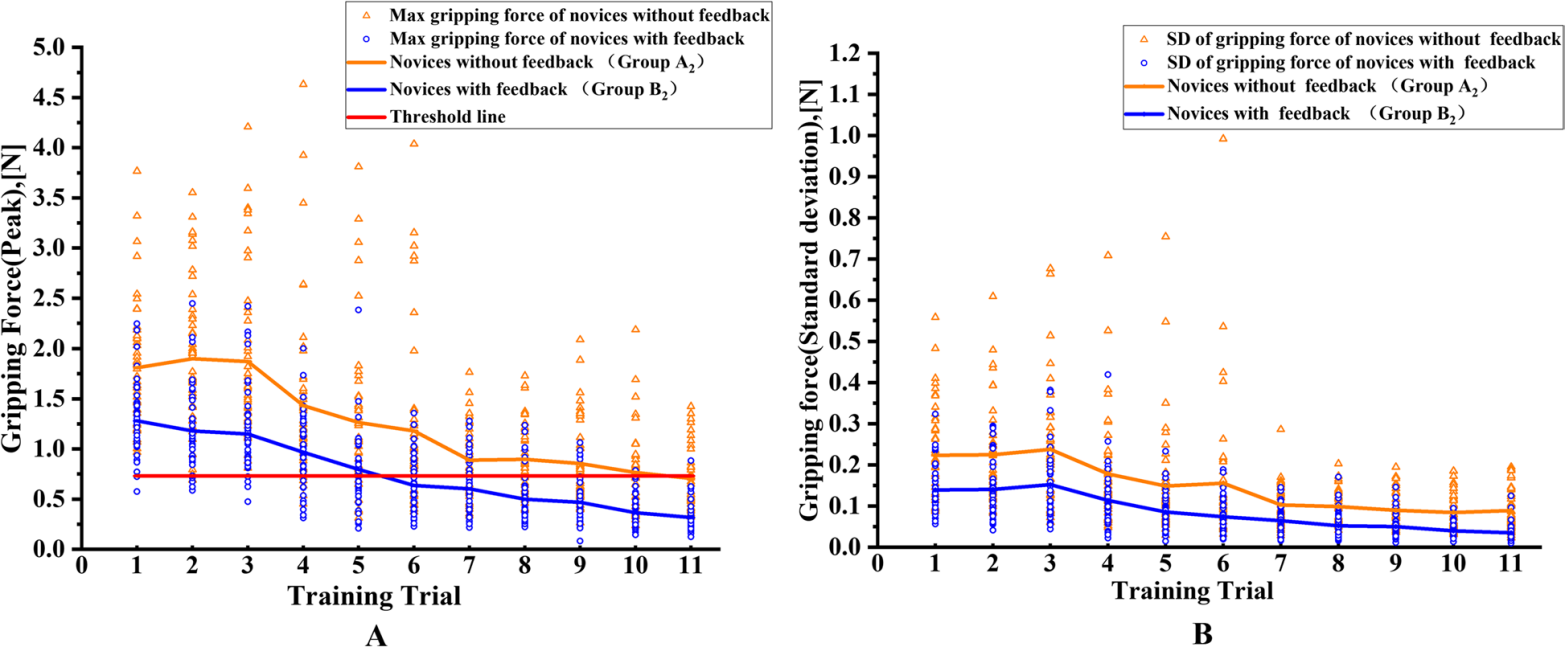
(**A**) and (**B**) represent the learning curves of maximum force and standard deviation of the gripping force, respectively (SD, standard deviation)

To gain a deeper understanding of the impact of force feedback on the learning curve, we presented the learning curves for the entire training process and subsequent follow-up trial. Figure 4 illustrates that in both groups, the maximum gripping force and standard deviation of the gripping force exhibit a gradual decrease throughout the training period.

In Fig. 4A, compared to Group A_2_, at the sixth training trial, the maximum gripping force of Group B_2_ is 0.635 N, which was below the threshold, while Group A_2_ still failed to reach the level below the threshold even by the tenth training. Furthermore, in the final training trial, the maximum gripping force of Group B_2_ was only 0.363 N, while the maximum gripping force of Group A_2_ is 0.765 N, still surpassing the threshold level. Obviously, Group B_2_ demonstrated a significantly shorter learning curve compared to Group A_2_. Therefore, the use of a smart grasper with an FBG force sensor can effectively expedite the training process and lead to a shorter curve.

In terms of the standard deviation of gripping force during training, which indicates gripping stability, Fig. 4B demonstrates that Group B_2_ exhibits a smaller standard deviation compared to Group A_2._ This implies that in the whole training process, Group B_2_ demonstrates superior stability and less fluctuation in gripping force than Group A_2_.

In the comparison of training effects in each stage of training stages (Table 5), the gripping force exhibited significant differences between Group A_2_ and Group B_2_ (*p<0.05*).These results indicate that novices who received feedback performed better during laparoscopic gripping training.

**Table 5:** Statistical results between different groups in training trials

Training Trial	Day 1	Day 5	Day 10	Day 11
Peak force	*p < 0.001*	*p < 0.001*	*p < 0.001*	*p < 0.001*
Standard deviation of force	*p < 0.001*	*p < 0.002*	*p < 0.001*	*p < 0.001*

Mann–Whitney *U* test

Significant *p* values are given in italics (*p* < 0.05)

### Analysis results of follow-up test

Throughout the follow-up period, there was no significant decline in force learning, and the gripping force continued to decrease. Without providing force feedback, the maximum gripping force of Group A_2_ was 0.706 N, while Group B_2_ was only 0.316 N. Notably, the maximum gripping force in both groups fell below the threshold level, indicating a better retention of force. Group B_2_ maintained superior gripping performance compared to Group A_2_ after training. Additionally, the standard deviation of the gripping force for Group A_2_ increased slightly to 0.089 N, whereas Group B_2_’s standard deviation continued to decrease to 0.035 N.

**Fig. 5:**
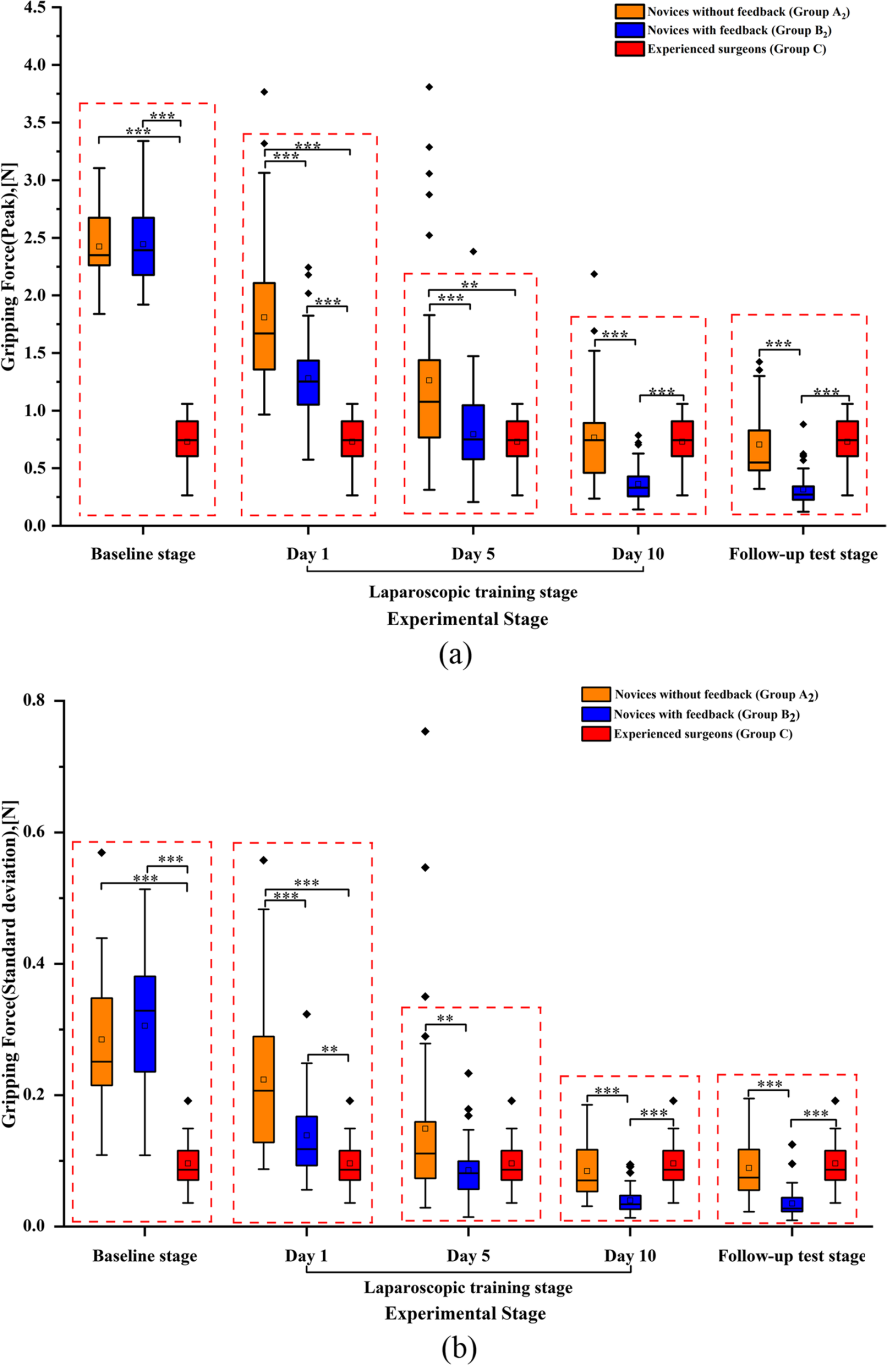
Gripping force at different stages under various conditions. (**a**) The maximum gripping force of Group C, Group A_2_ and Group B_2_ during baseline assessment stage, training stage and follow-up stage, (**b**) the standard deviation of gripping force of Group C, Group A_2_ and Group B_2_ during these three stages

In Fig. 5, we employed statistical methods to analyze the impact of force feedback on laparoscopic training across the baseline stage, training stage, and follow-up stage. During the baseline stage, the results indicate that there were no statistically significant differences in both the maximum and standard deviation of gripping force between Group A_2_ and Group B_2_(*p*>0.05). These findings suggest that novices in both groups performed at a similar level of gripping proficiency. Compared to the experienced surgeons, novices tend to apply higher gripping force during the initial stage of an operation. This often causes unnecessary tissue damage in clinical operation, so it is essential to train novices and verify whether incorporating force feedback in the operation has a positive effect on novices’ training. During the training phase, the results showed that the maximum force and standard deviation of the novices decreased during the training process. Moreover, the maximum gripping force of Group B_2_ on day 1, 5 and 10 were 1.281 N, 0.796 N, 0.363 N respectively while they were 1.811 N, 1.263 N, 0.765 N of Group A_2._ The difference between Group A_2_ and Group B_2_ was statistically significant (*p* < 0.05), indicating that the training effect was better with providing force feedback. Without providing force feedback, novices completed the follow-up period. The maximum gripping force of Group A_2_ was 0.706 N, while Group B_2_ was only 0.316 N. The difference between Group A_2_ and Group B_2_ was statistically significant (*p* < 0.05), indicating that Group B_2_ maintained superior gripping performance compared to Group A_2_ after training.

All thirty novices enrolled in the training period completed the questionnaire. The evaluation scales for both the force feedback system and force feedback training utilized a 5 points Likert scale, which a maximum score of 5 points per question. As illustrated in Table 6, the system design received a score of 4.53 ± 0.52, indicating high satisfaction with the system. Similarly, regarding the visualization, instrumentation, user-friendliness, task description, force accuracy of feedback, and necessity of the system, scores were closed to the maximum of 5 points. Regarding for the evaluation of the force feedback training, participants self-rated an improvement in their technical skills and self-confidence as a result of the training. Moreover, trainees expressed that they found the training to be necessary and stated their willingness to recommend it to others. In term of the results from The NASA Task Load Index (Table 7), which assesses novices’ subjective feeling during the training, it was observed that novices who received feedback obtained lower scores in mental stress, psychological burden, operating time, and effort compared to those without feedback. These results suggest that the use of the smart laparoscopic grasper, which provides real-time force feedback, can lead to a more comfortable and confident gripping experience for novices. When it comes to the feeling of satisfaction, the scores of novices with feedback was higher than those without feedback_,_ indicating that the incorporation of feedback resulted in a more positive emotional experience during the training.

**Table 6:** Survey outcomes

Statement	5 points Likert scale Presented as mean (±SD)
Training feedback system	
The system design	4.53 ± 0.52
Screen/visualization	4.73 ± 0.46
The instrument	4.40 ± 0.51
User friendliness	4.60 ± 0.51
Mission statement	4.87 ± 0.35
The feedback force differs little from the actual applied force	4.40 ± 0.63
This training system is necessary	4.67 ± 0.49
Training feedback	
The training is useful	4.70 ± 0.47
Skills develop through training	4.73 ± 0.52
Confidence goes up	4.43 ± 0.73
Such training is necessary	4.63 ± 0.56
I would like to recommend this training to others	4.77 ± 0.43

**Table 7:** The NASA task load index

The NASA Task Load Index	Mean score (±SD)		
	All subjects (*n* = 30)	Novices without feedback (n = 15)	Novices with feedback (*n* = 15)
Mental Demands: How much mental effort is required to complete a task?	43.67 (±17.90)	50.00 (±18.90)	37.33 (±14.86)
Physical demands: How much or little is the physical burden of completing the task?	31.00 (±16.68)	32.67 (±17.10)	29.33 (±16.68)
Time requirements: Do you complete tasks slowly or flustered?	47.67 (±25.96)	58.00 (±26.78)	37.33 (±21.20)
Effort: How much or how little effort did you put into completing the task?	43.67 (±21.73)	46.00 (±21.97)	41.33 (±22.00)
Frustration: Do you feel safe/gratified/satisfied/relieved?	66.33 (±32.32)	59.33 (±34.74)	73.33 (±28.95)

*SD* Standard deviation

## Discussion

This study included a preliminary experiment to evaluate the impact of real-time force feedback on gripping, which demonstrated improved control of gripping force during MIS. Furthermore, a four-week laparoscopic training was conducted to confirm that force feedback helps to shorten the learning curve in laparoscopic training. Through the use of proposed smart laparoscopic grasper integrated with an FBG-based tactile sensor, novice participants were able to achieve real-time force feedback resulting in a shorter learning curve and an improved control of the gripping force during training. As a further assessment, a follow-up gripping operation was carried up, and several scales were performed to gauge the participants’ subjective perception of the training process. During the follow-up period, novices who had trained with force feedback demonstrated improved control of gripping even without the provision of force feedback. As for the result of scales assessment, both the training system itself and the emotional experience of the training process, as well as the benefits derived from the training, were highly rated. Accounting for the novices may vary in different gripping levels at the beginning, which somehow influence the result of the study, we did a baseline assessment and only participants at the same level were enrolled in the experiment. While individual abilities may differ, there were no significant differences in the overall evaluation. This suggests that the shortened learning curve is mainly due to the use of force feedback instead of the differences in individual participants’ abilities. Besides, taking the schedule of the novices into consideration, we ensured that all novices had the same number of gripping trails during the four-week training. Novices were allowed to take their time to complete the gripping tasks, accommodating their individual pace.

As pointed out by Hopper et al. [34], the ideal surgical learning curve (LC) tends to show a steep curve at first, which then fades out to a more gradual LC as the plateau phase approached. The technical skills are sufficiently established to operate independently and safely at last [35]. In our study, both the maximum force and the standard deviation of force were observed to have significantly decreased throughout the training period, as shown in Fig. 4. Furthermore, during the follow-up period, there was no significant increase in gripping force. On the contrary, gripping force further decreased due to improved control of the grasper. These results confirmed that the training effect can be preserved, and the LC of novices reach a fairly stable stage. Furthermore, compared to novices without feedback (Group A_2_), novices with feedback (Group B_2_) were observed to achieve better control of gripping force during the training. The learning curve of Group B_2_ was also noticeably shortened in comparison to Group A_2_. These results further demonstrate the effectiveness of real-time force feedback in improving the learning process and enhance the performance of novices in laparoscopic training. According to the results of our study, the gripping skills in the training box of the novices are improved because of the training and real-time force feedback. The integration of a smart laparoscopic grasper with force feedback capability contributed to maintaining optimal gripping force levels and results in a shorten the learning curve. These results highlight the potential benefits of incorporating force feedback technology in laparoscopic training to improve the performance and skill acquisition of novices. The introduction of force feedback is a feasible and valuable mechanism for enhancing laparoscopic training among novices. In the future, it should be considered as an integrated part of laparoscopic training programs, allowing for the development of individualized courses based on trainee’s learning curve. Eventually, the cost of laparoscopic learning training is expected to decrease. Besides, implementing a threshold for gripping tasks serves to raise novice’s awareness about the potential risks associated with applying excessive forces in the box trainer, which can lead to unnecessary tissue damage. Real-time force feedback allows them to adjust their gripping force in time according to the threshold, which is likely to be beneficial in clinical surgery [36]. If laparoscopy novices can hand and adjust their force in time, tissue damage [37] will be reduced. Furthermore, by analyzing the learning curve of the novices in the laparoscopic training, we can distinguish skill levels of surgeons to some extent [38]. Thus, a more targeted and individualized training plan can be established to realize precise training [39]. The promising results demonstrate the feasibility and value of integrating force feedback into laparoscopic training for novices. This study highlights the significant potential of the smart laparoscopic grasper with FBG force sensor in improving training, quality and reducing the learning curve. Ultimately, it offers a robust force feedback system for MIS.

In summary, the results of our study show that the utilization of an intelligent laparoscopic grasper with real-time force feedback in laparoscopic training contributes to improve the control of gripping force and a shortened learning curve.

A few limitations are encountered in the research. First of all, two screens may affect the mental and cause distraction in the process of gripping. The real-time force feedback of the training system needs to be further optimized by setting an operational screen in a form of “traffic lights” [40] or audio reminders. Secondly, clinical operations involve multiple gripping motion, and training tasks should be designed to imitate these movements. Finally, in-vivo experiments will be necessary to determine whether the positive effects observed in our study can be transferred to real-time scenarios. While our current results are not based on in-vivo testing, they provide promising evidence of the potential benefits of incorporating force feedback into laparoscopic training.

## Conclusion

In conclusion, using a grasper providing real-time force feedback in laparoscopic training can help to control the gripping force and shorten the learning curve. It is anticipated that the laparoscopic grasper equipped with fiber Bragg grating sensor is promising to provide force feedback during laparoscopic training, which ultimately demonstrating significant potential in the field of laparoscopic surgery.

## Data Availability

The datasets used and analyzed during the current study are available from the corresponding author on reasonable request.

## Abbreviations

MIS: Minimally Invasive Surgery
RMIS: Robot-assisted Minimally Invasive Surgery
BT: Box Training
VR: Virtual Reality
AR: Augmented Reality
FBG: Fiber Bragg grating
EMI: Electromagnetic Interference
LC: Learning Curve
